# Epothilone D alters normal growth, viability and microtubule dependent intracellular functions of cortical neurons *in vitro*

**DOI:** 10.1038/s41598-020-57718-z

**Published:** 2020-01-22

**Authors:** J. A. Clark, J. A. Chuckowree, M. S. Dyer, T. C. Dickson, C. A. Blizzard

**Affiliations:** Menzies Institute for Medical Research, University of Tasmania 17 Liverpool Street Hobart, Tasmania, 7000 Australia

**Keywords:** Cellular neuroscience, Diseases of the nervous system

## Abstract

Brain penetrant microtubule stabilising agents (MSAs) are being increasingly validated as potential therapeutic strategies for neurodegenerative diseases and traumatic injuries of the nervous system. MSAs are historically used to treat malignancies to great effect. However, this treatment strategy can also cause adverse off-target impacts, such as the generation of debilitating neuropathy and axonal loss. Understanding of the effects that individual MSAs have on neurons of the central nervous system is still incomplete. Previous research has revealed that aberrant microtubule stabilisation can perturb many neuronal functions, such as neuronal polarity, neurite outgrowth, microtubule dependant transport and overall neuronal viability. In the current study, we evaluate the dose dependant impact of epothilone D, a brain penetrant MSA, on both immature and relatively mature mouse cortical neurons *in vitro*. We show that epothilone D reduces the viability, growth and complexity of immature cortical neurons in a dose dependant manner. Furthermore, in relatively mature cortical neurons, we demonstrate that while cellularly lethal doses of epothilone D cause cellular demise, low sub lethal doses can also affect mitochondrial transport over time. Our results reveal an underappreciated mitochondrial disruption over a wide range of epothilone D doses and reiterate the importance of understanding the dosage, timing and intended outcome of MSAs, with particular emphasis on brain penetrant MSAs being considered to target neurons in disease and trauma.

## Introduction

Historically, the adult neuronal cytoskeleton was considered relatively stable, being involved only in neuroprogenitor cell division during de novo neurogenesis^1,2^. With our increased understanding of the neuronal microtubule environment and function, and the adult brains capacity for neuroplasticity^3^, it is now evident that neuronal microtubules, the largest of the three filament components of the cytoskeleton, form a critical dynamic intracellular network^4^. In this regard, microtubules consist of both stable and labile domains^5,6^ within tubular protofilaments comprised of α- and β-tubulin heterodimers. Microtubules display a phenomenon termed “dynamic instability”^7^, which describes the stochastic variations between bouts of growth, pausing and shrinkage of the microtubule population. Microtubules can transition from a growing to a shrinking state, termed catastrophe, which may or may not be rescued back to a growing state. The microtubule protofilament consists of a slow growing “minus end” and a fast growing, highly dynamic “plus end”^8,9^, which plays a critical role in normal neuronal functions including neuronal growth, intracellular transport, establishment and maintenance of cellular polarity, and process remodelling^10–12^. Changes to the growth state of microtubules can have downstream consequences for their normal physiological functions, such as the binding of specific motor transport units^13^, and the association of + TIP proteins that facilitate interactions between microtubules and other intracellular structures, and regulate important neuronal outcomes such as axonal branching^14,15^.

Manipulation of microtubule dynamics and structural integrity can be accomplished by using compounds termed ‘microtubule stabilising agents’ (MSAs). At high doses, MSAs are among the most successful means by which to target and treat various types of malignancies^16^. This is mainly due to the importance of microtubules in the development and mechanical action of the mitotic spindle during cell division, which progresses into an uncontrolled and hyper activated process in cancer^17^. Unfortunately, this treatment strategy has ramifications for otherwise healthy cells of the body. In neurons, abolishing microtubule dynamics causes painful peripheral neuropathies; an adverse effect associated with chemotherapy treatment in cancer patients (reviewed previously in^18,19^). This is particularly prevalent in sensory neurons, where MSAs cause microtubule protein alterations, microtubule disintegration and axonal degeneration^20^. In this regard, MSAs have been shown to induce aberrant shifts in the polarity of axonal microtubules from a “plus end out” uni-directional orientation, to a mixed orientation similar to that of dendrite microtubules. Ultimately this altered orientation impacts on microtubule based transport^21^. MSAs have also been documented to influence the cortical circuitry of the central nervous system (CNS). For example, low doses of the MSA taxol have been shown to alter the dynamics of dendritic spines^22^, and can augment memory formation^23^.

This identification of positive effects of MSAs on the nervous system has led to an explosion of research involving the use of brain penetrant MSAs to treat various neurodegenerative disorders of the CNS^24^. A family of MSAs currently utilised in many studies are the epothilones, a group of compounds found naturally in the myxobacterium *Sorangium cellulosum*^25^. Previous investigations report a dose dependant response to epothilone D (EpoD) treatment after a scratch injury in cortical neurons *in vitro*, with low micromolar to subnanomolar concentrations of EpoD increasing axonal sprouting post-injury^26^. Others have reported positive results when utilising EpoD to improve outcomes in *in vivo* models of Parkinson’s Disease^27^, tauopathy and Alzheimer’s Disease^28,29^, and schizophrenia^30,31^. However, a recent study reports that low doses of the epothilone analogue epothilone B cause alterations to neuronal viability and growth^32^. Collectively, these studies indicate that epothilones may have an unappreciated dose dependant range of outcomes.

Concerns regarding the therapeutic use of MSAs centres around the lack of understanding of individual brain penetrant MSAs and their dose dependent effect on neuronal health in the CNS^33^. Indeed, investigations into the impact EpoD has on neuronal viability, growth and function are yet to be completed in cortical neurons, a neuronal population increasingly targeted by MSAs. In the current study we aimed to directly address this shortfall by identifying the toxic dose range of EpoD *in vitro* using cortical neuron cultures. We proposed that EpoD would provoke dose dependant alterations to outcome measures, such as cortical neuron survival, growth and complexity, alterations to microtubule associated protein expression and microtubule dependant organelle transport. Using a mouse primary cortical neuron culture system, we report novel findings of neuronal dysfunction due to EpoD treatment *in vitro*, with implications for future studies utilising low-dose antimitotic compounds, such as EpoD, for the treatment of neurodegenerative disorders.

## Methods

### Animals

Animal use was approved by the Animal Ethics Committee of the University of Tasmania and was performed in accordance with the ‘Australian Code of Practice for the Care and Use of Animals for Scientific Purposes (2013)’. All animal experiments utilised transgenic yellow fluorescent protein (YFP) mice (*thy*1-YFP-16Jra/J; stock number 003709) (The Jackson Laboratory), which express YFP in large projection neurons (namely pyramidal cells) in the CNS^34^. All mice were maintained on a C57Bl/6 background.

### Primary cortical neuron cultures

Embryonic cortical neurons were isolated from the dissected and dissociated neocortex of 15.5-day-old embryonic YFP mice and plated onto a poly-l-lysine coated surface (glass coverslips etched in 70% nitric acid or plastic) in 24 well plates (1.9 cm^2^), 6 well plates (3.48 cm^2^) or live imaging micro chambers (1.5 cm^2^). Neurons were maintained in neurobasal medium (Thermo Fischer Scientific), containing 2% B27 supplement (Thermo Fischer Scientific), L-glutamine (Sigma) and 1x penicillin-streptomycin (Thermo Fischer Scientific), and incubated at 37 °C in 5% CO_2_ for the duration of experiments.

### Treatment paradigms

In order to study the effects of EpoD (ABCAM, stock number ab143616, >99% purity) on normal neuronal development, neurite growth and function, cells were exposed to a variety of drug concentrations (0.1 nM, 1 nM, 10 nM, 100 nM EpoD), for two hours and 24 hours in relatively mature neurons (10DIV), or 24 hours with media replacement (conditioned media plus treatment) for growth and development experiments from one to four DIV. Naïve, untreated neurons and vehicle (0.1% *v/v* DMSO in culture media) treated neurons were utilised as controls. All experiments used a minimum of three separate cultures, with experiments completed in triplicate, unless otherwise stated.

### Quantification of cell viability

**Figure 1:**
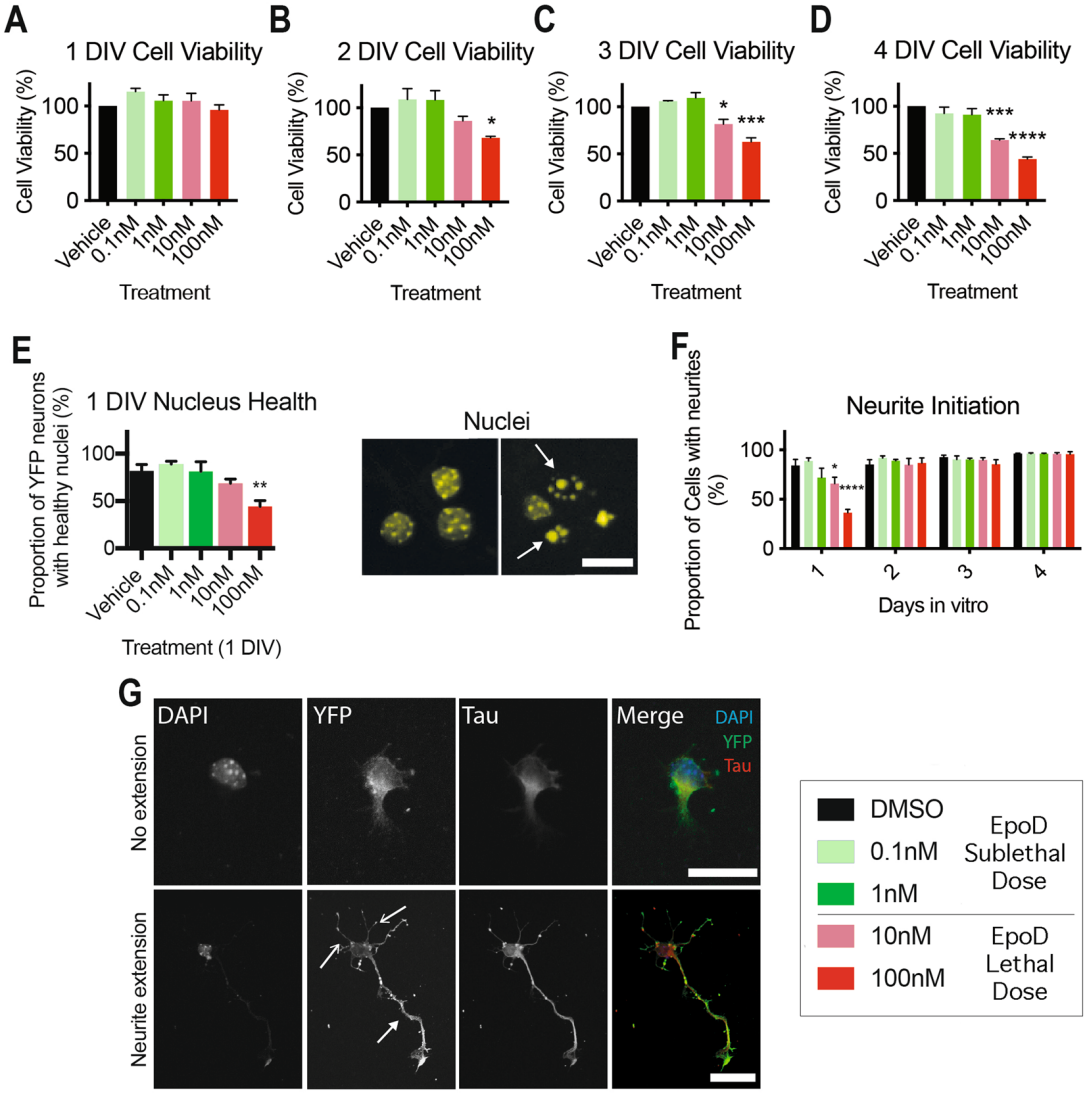
Neuronal viability and neurite process extension of EpoD treated immature cortical neurons. (**A**) There is no decrease in cell viability at 1DIV. (**B**) At 2DIV 100 nM EpoD treated cortical neurons have a significant (*p* < 0.05) decrease in cell viability. (**C**) 10 nM and 100 nM EpoD treated cortical neurons exhibit a significant (*p* < 0.05) reduction in cell viability at 3DIV, and (**D**) further reduction (*p* < 0.05) in viability of 10 nM and 100 nM treated cultures at 4 DIV. (**E**) The proportion of YFP positive cells with healthy nuclei is significantly reduced (*p* < 0.001) in 100 nM EpoD treated cultures at 1DIV. (**F**) The proportion of healthy YFP positive neurons with neurites are significantly (*p* < 0.05) reduced in 100 nM EpoD treated cortical neuronal cultures at 1DIV, but is comparable to vehicle treated control cultures by 2, 3 and 4DIV. (**G**) Immunocytochemistry example of a healthy YFP positive cortical neuron with no process extension (top images) and a YFP positive cortical neuron with an extending tau positive process (bottom images, solid arrowhead) and shorter, more numerous tau negative processes (open arrowheads). Using the 3–4 DIV cell viability assays, EpoD concentrations were classified as either sublethal (did not cause loss of cell viability) or lethal (caused significant loss in cell viability. Scale = 90 μm. (Data reported as mean ± SD, ANOVA with Dunnett post hoc test, *p < 0.05, ***p < 0.001. All data *n* = 3 cultures, performed in triplicate. Abbreviations: DIV, days *in vitro*; EpoD, epothilone D; SEM, standard error of the mean; nM, nanomolar; YFP, yellow fluorescent protein. *p < 0.05, **p < 0.01, ***p < 0.001.

Cell health was determined by using the AlamarBlue® cell viability assay (Thermo Fisher Scientific) according to the manufacturer’s instructions and measured by fluorescence on a FLUOstar OPTIMA plate reader (excitation 570 nm, emission 580; BMG Labtech). Data are reported as percentage of cell viability corrected to vehicle treated controls. The AlamarBlue® cell viability assay was complemented with the evaluation of nuclear morphology using DAPI staining, to determine cell viability, as described previously^35^. Briefly, nuclei were graded as “healthy” when DAPI labelling could determine the nuclear boundary, with diffuse staining throughout the nucleus (see Fig. 1E); or graded as “unhealthy/dying” if nuclei appeared pyknotic/fragmented, with no definable nuclear boundary (see Fig. 1E, arrows).

### Immunocytochemistry and confocal microscopy

Neuronal cultures were fixed in 4% PFA, followed by permeabalisation for 10 minutes (0.3% or 1% Triton-x-100 in 0.01 M PBS containing 5% BSA) and then incubated in primary antibody in 0.01 M PBS containing 5% BSA overnight at 4 °C. Antibodies were used to identify cell processes (rat-monoclonal GFP, 1:3000, Nacalai Tesque), axons (rabbit-polyclonal tau, 1:2000, DAKO). Neurofilaments were labelled with antibody to neurofilament medium chain (NFM) (rabbit-polyclonal NFM, 1:1000, Millipore). Secondary antibodies (anti-rat Alexa488, anti-rat Alexa594, anti-mouse Alexa568, anti-rabbit Alexa647) were diluted in PBS (1:750; Molecular Probes) and cultures were incubated for 1.5 hours at room temperature, followed by DAPI nuclear staining, washing with PBS, and mounting with mounting media (Permafluor, DAKO). Cells were visualised using a spinning disk laser confocal (UltraVIEW® VOX, Perkin Elmer) on an inverted microscope (Nikon TiE, Nikon) fitted with 20 × (Plan-Apo N.A. 0.75) and 40 × (Plan-Apo N.A. 0.95) objective lenses. Images were acquired using an Orca R^2^ camera (C10600, Orca, Hamamatsu) and processed using image capture software (Velocity v6.3.0, 2013, Perkin Elmer).

### Image analysis and cell tracing

Neurite extension was evaluated in YFP expressing cells with healthy nuclear morphology and robust YFP cytoplasmic fluorescence in Image J (NIH)^36^. Tracings of neuronal cell bodies, developing axons (tau positive YFP processes) and dendrites (tau negative YFP processes) were completed utilising Neurolucida software (MBF Bioscience). The area of Neurofilament-M (NFM) and YFP immunostaining was evaluated by firstly thresholding images using settings determined from vehicle treated cultures, followed by particle analysis to calculate staining area in ImageJ. To determine the level of NFM in YFP neurons specifically, a Coloc 2 plugin was used to determine the Pearson’s correlation coefficient between NFM and YFP. The immunostaining area change and correlation coefficient is represented as a ratio to vehicle treated controls.

### Mitochondrial transport analysis

Culture media containing EpoD/vehicle treatment was removed from the cells and kept at 37 °C for reapplication following mitochondrial staining. Cells were incubated in conditioned culture media containing Mitotracker® Red FM (200 nM final concentration, Thermo Fischer Scientific) for 15 minutes, followed by a 5 minute wash in conditioned culture media. EpoD/vehicle media was reapplied to the cells and live cell imaging was performed utilising fluorescence, differential interference contrast (DIC) and phase-contrast microscopy on a Nikon Eclipse Ti inverted microscope (Nikon) within an enclosed incubator maintained at 37 °C and 5% CO_2_. Live cell images were captured using a Zyla CMDS camera (Andor), and controlled by NIS elements (Nikon) (two minutes, one second intervals). Images were subsequently analysed using FIJI software (NIH), with the plugin MTrackJ^37^. Moving mitochondria were traced until they no longer moved for three or more frames, or until the end of the imaging session. Data from mitochondria with multiple movement phases was averaged.

### Western blot protein quantification

Neuronal cultures were rinsed in ice-cold PBS, followed by lysis in RIPA-Inhibition buffer (RIPA buffer + 1 μM trichostatin-a (Sigma Aldrich) + protease inhibitor cocktail (Sigma Aldrich)), and centrifugation at 15,000 g for 10 minutes at 4 °C to remove cell debris, as described previously^28^. Proteins were separated by SDS-PAGE using a Novex NuPAGE gel system (Thermo Fisher Scientific), followed by probing by Western blot. Protein levels were then evaluated using antibodies to acetylated tubulin (mouse-monoclonal, 1:5000, Sigma Aldrich), alpha-tubulin (mouse-polyclonal, 1:5000, Abcam), MAP2 (mouse-monoclonal, 1:1000, Millipore, MAB3418), MAP6/STOP (mouse-monoclonal, 1:1000, Millipore, MAB5524) and EB3 (rat-monoclonal, 1:200, Abcam, AB53360), with GAPDH (rabbit polyclonal, 1:5000, Millipore) and alpha-tubulin (mouse-monoclonal, 1:5000, Abcam) used as loading controls. Secondary HRP conjugated antibodies (DAKO anti-mouse and rabbit, 1:5000; Invitrogen anti-rat, 1:2000) were incubated for 1.5 hours, followed by rinsing with TBS-T. Membranes were then probed by incubating with Immobilon chemiluminescence HRP substrate (Millipore) for 5 mins, followed by detection with Chemi-Smart 5000 image station (Vilber Lourmat). Band quantitation was completed using densitometry analysis in ImageJ. Blot stripping was completed using Restore Western Blot Stripping Buffer (Thermo Fisher Scientific, 21059).

### Statistical analysis

Neuronal growth, development and function was statistically analysed using GraphPad Prism (Version 5.00, La Jolla, CA), with data representing percentage change from vehicle controls undergoing ‘remove baseline’ functions. All data was compared to vehicle using one-way analysis of variance (ANOVA) with Dunnets post-hoc correction for multiple comparisons. *p* < 0.05 was considered significant. Data is expressed ± standard error of the mean (SEM) and normalised to vehicle controls. Unless otherwise stated, a minimum of three separate cultures was used for each experiment, with three technical replicates per culture (*n* = 3).

## Results

### Epothilone D induces cellular toxicity and limits neuronal maturation in a dose and time dependant manner

Pharmacological stabilisation of microtubules is associated with neurotoxicity and microtubule dependent neuronal dysfunction^20,21^. We hypothesised that EpoD would have dose dependant effects on neuronal viability, development and survival *in vitro*. To assess the effect of microtubule stabilisation on neuronal viability and the capacity for neurite extension, we performed an AlamarBlue® cell viability assay, nuclear morphology analysis and evaluation of neurite extension of immature embryonic YFP positive mouse cortical neurons. EpoD was administered to developing cultures by bath application in concentrations of 0.1 nM, 1 nM, 10 nM and 100 nM, which was replaced at 24 hour time intervals for 4 days^28,38,39^.

Developing neurons treated with varying doses of EpoD showed no significant (*p* > 0.05) changes in neuronal viability compared to vehicle treated control neurons at 1DIV (Fig. 1A). At 2DIV, 100 nM EpoD treated cultures showed significantly (*p* < 0.05) reduced cell viability by 32%, vehicle treated control neuron (Fig. 1B).

By 3DIV (two days of recurrent treatment), the percentage viability was significantly (*p* < 0.01 and *p* < *0.001*) reduced by 18% in 10 nM EpoD treated cultures and 37% in 100 nM EpoD treated cultures, compared to vehicle treated controls (Fig. 1C). Cell viability decreased further, to 35% in 10 nM EpoD treated cultures and 56% in 100 nM EpoD, when compared to vehicle treated controls (Fig. 1D), suggesting that higher doses of EpoD are detrimental to neuronal cell growth and survival.

Evaluation of nuclear morphology^35^ was also used as a readout for gross EpoD toxicity in neurons, to better estimate the lethal dose range *in vitro*. YFP positive neurons showed a significant (*p* < 0.05) 38% decrease in healthy nuclei in cultures treated with 100 nM of EpoD for 24 hours (1DIV), compared to a age matched vehicle controls (Fig. 1E). This data suggests that we have selected two sublethal doses (0.1 nM and 1 nM) and two lethal doses (10 nM and 100 nM) of EpoD, as indicated in the legend of Fig. 1, and will be colour coded as such henceforth.

Previous reports demonstrate that correct microtubule function is necessary for neurite extension during neuronal development^40^. Neurite outgrowth was evaluated in YFP positive neurons with healthy nuclear morphology and robust YFP positive cytoplasmic volume (Fig. 1F,G). A large proportion of vehicle treated neurons presented with neurite extensions by 1DIV (Fig. 1F,G), whereas both lethal 10 nM and 100 nM EpoD treated cultures showed a significant (*p* < 0.05 and *p* < 0.001) 18% and 48% decrease in the proportion of neurons with neurite extensions compared to vehicle treated controls, respectively. By 2, 3 and 4DIV, the proportion of neurons with neurites was comparable to vehicle controls (Fig. 1F), suggesting that high lethal doses of EpoD inhibit initial neurite outgrowth in primary cortical neurons; however, surviving neurons are able to sprout processes at later stages of development, irrespective of EpoD concentration.

### The impact of epothilone D on neurite complexity in primary cortical neurons

**Figure 2:**
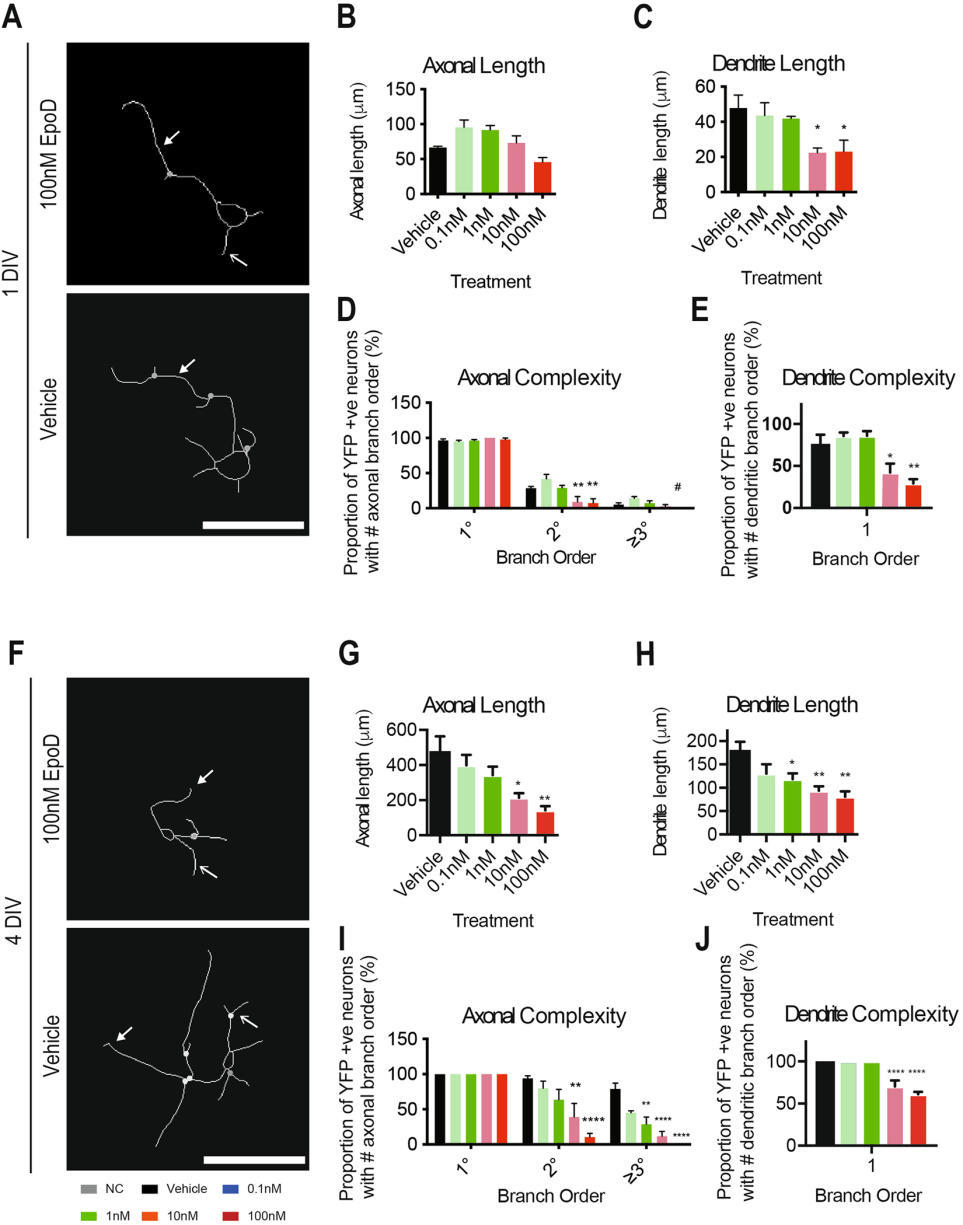
Alterations to neurite growth and complexity in EpoD treated immature cortical neurons. (**A**) Example tracings of YFP neurons at 1DIV show decreased neurite complexity when treated with EpoD, compared to vehicle treated neurons (tau positive axonal process, closed arrowhead; tau negative dendritic process, open arrowhead). Scale = 45 μm. (**B**) Total length of axonal processes are not significantly (*p* > 0.05) altered due to EpoD treatment, (**C**) however, the total length of dendritic processes are significantly (*p* < 0.05) decreased at higher doses of EpoD. Data reported as mean ± SEM, ANOVA with Dunnett post-hoc test. (**D**) The proportion of secondary order axonal processes is significantly (*p* < 0.05) decreased in 10 nM and 100 nM EpoD treated YFP neurons and similarly, (**E**) the proportion primary order dendritic processes is significantly (*p* < 0.01) reduced in 10 nM and 100 nM EpoD treated YFP neurons. Data reported as mean ± SEM, ANOVA with Tukey’s post hoc-test. (**F**) Example tracings of YFP neurons at 4DIV show decreased neurite complexity when treated with EpoD, compared to vehicle treated neurons (tau positive axonal process, closed arrowhead; tau negative dendritic process, open arrowhead). Scale = 75 μm. (**G**) Total length of axonal processes are significantly (*p* < 0.001) decreased in 10 nM and 100 nM EpoD treated YFP neurons. (**H**) Total length of dendritic processes are significantly (*p* < 0.05) decreased in 0.1 nM, 10 nM and 100 nM EpoD treated YFP neurons. Data reported as mean ± SEM, ANOVA with Dunnett post hoc-test. (**I**) The proportion of secondary order axonal processes is significantly (*p* < 0.001) decreased in 10 nM and 100 nM EpoD treated YFP neurons. Tertiary order processes are also significantly decreased (*p* < 0.01) in 1 nM and 10 nM EpoD treated YFP neurons, with no tertiary order axonal process in 100 nM treated cells. (**J**) The proportion of primary order dendritic processes is significantly (*p* < 0.05) reduced in 10 nM and 100 nM EpoD treated YFP neurons. Circles in example traces re*p*resent branch points. Data reported as mean ± SD, ANOVA with Tukey’s post hoc-test. All data n = 4 cultures, performed in triplicate. Abbreviations: DIV, days *in vitro*; EpoD, epothilone D; nM, nanomolar; SEM, standard error of the mean; YFP, yellow fluorescent protein. *p < 0.05, **p < 0.01, ***p < 0.001, ****p < 0.0001.

As well as being important for neurite initiation and the induction of neuronal polarity, microtubules are also important for axonal and dendrite growth and the development of circuit complexity^41,42^. To determine whether EpoD treatment has microtubule dependant effects on these parameters, developing YFP positive cortical neurons were treated with varying doses of EpoD, followed by evaluation of tau positive (axon henceforth) and tau negative (dendrites henceforth) neurite process complexity at 1DIV and 4DIV. Treatment with 10 nM and 100 nM EpoD was found to decrease the complexity of YFP cortical neurons at 1DIV, compared to vehicle treated neurons (Fig. 2A). Although there was a subtle increase in axonal lengths in 01.nM and 1 nM EpoD treated cultures, this was not significant (*p* > 0.05) when compared to vehicle treated controls at 1DIV (Fig. 2B). However, dendritic processes at 1DIV were significantly (*p* < 0.05) shorter in both 10 nM and 100 nM EpoD treated YFP cortical neurons, compared to vehicle treated controls (10 nM EpoD: 20.95 μm ± 4.75 μm; 100 nM EpoD: 23.08 μm ± 9.14 μm; vehicle: 41.81 μm ± 15.97 μm) (Fig. 2C). The complexity of secondary order axonal processes was significantly reduced in 10 nM and 100 nM EpoD treated neurons, compared to vehicle controls at 1DIV, with no 100 nM treated neurons containing tertiary order process at 1DIV (10 nM EpoD: 9.14% ± 15.14%; 100 nM EpoD: 7.57% ± 11.88%; vehicle: 28.41% ± 4.87%) (Fig. 2D). Similarly, the proportion of YFP neurons with primary order dendritic processes was also significantly decreased in 10 nM and 100 nM EpoD treated cultures (10 nM EpoD: 42.17% ± 21.14%; 100 nM EpoD: 29.26% ± 10.11%; vehicle: 76.47% ± 21.51%) (Fig. 2E). There was no significant (*p* > 0.05) difference in the complexity of dendritic secondary and tertiary processes due to EpoD treatment at 1DIV (data not shown).

Similar to treatments at 1DIV, 10 nM and 100 nM concentrations of EpoD were found to reduce the complexity of cortical neurons at 4DIV, compared to vehicle treated neurons (Fig. 2F). YFP neurons treated with 10 nM and 100 nM EpoD for four days have a significantly reduced (*p* < 0.05) total axonal process length, compared to vehicle treated controls (10 nM EpoD: 214.24 μm ± 44.15 μm; 100 nM EpoD: 141.81 μm ± 42.69 μm; vehicle: 479.31 μm ± 145.60 μm) (Fig. 2G). Similarly, dendritic processes exhibit significantly (*p* < 0.05) reduced total process length when treated with 0.1 nM, 10 nM and 100 nM of EpoD, compared to vehicle treated controls (10 nM EpoD: 93.81 μm ± 16.18 μm; 100 nM EpoD: 80.87 μm ± 19.32 μm; vehicle: 170.49 μm ± 15.59 μm) (Fig. 2H). Branching complexity at 4DIV in YFP neurons revealed that primary order axonal processes were not significantly (*p* > 0.05) altered due to EpoD treatment (Fig. 2I). However, secondary order axonal processes were significantly (*p* < 0.01) decreased in 10 nM and 100 nM EpoD treated YFP neurons, compared to vehicle treated controls (10 nM EpoD: 38.91% ± 38.87%; 100 nM EpoD: 10.23% ± 11.16%; vehicle: 93.75% ± 7.98%) (Fig. 2I). Tertiary order axonal processes were also significantly (*p* < 0.001) decreased in 1 nM and 10 nM EpoD treated YFP neurons, with 100 nM EpoD treated YFP neurons exhibiting no tertiary order processes (1 nM EpoD: 28.66% ± 20.74%; 10 nM EpoD: 11.52% ± 14.28%; vehicle: 78.89% ± 17.00%) (Fig. 2I). Evaluation of branching complexity of dendritic processes at 4DIV showed a significant (*p* < 0.001) decrease in the proportion of primary processes in 10 nM and 100 nM EpoD treated YFP neurons, compared to vehicle treated controls (100 nM EpoD: 60.96% ± 6.14%; vehicle: 100% ± 0%) (Fig. 2J). However, there was no significant (*p* > 0.05) decrease in the proportion of secondary processes in EpoD treated YFP neurons (data not shown). These results suggest that EpoD impacts on the growth and complexity of both axons and dendrites, with sub lethal 1 nM, and lethal 10 nM and 100 nM concentrations of EpoD impeding neurite outgrowth in YFP cortical neurons. It also suggests that although 10 nM and 100 nM concentrations of EpoD decrease cell viability and complexity, a subset of cortical neurons are able to develop complex neurite architecture.

### Epothilone D alters microtubule associated proteins involved in microtubule regulation and stability

**Figure 3:**
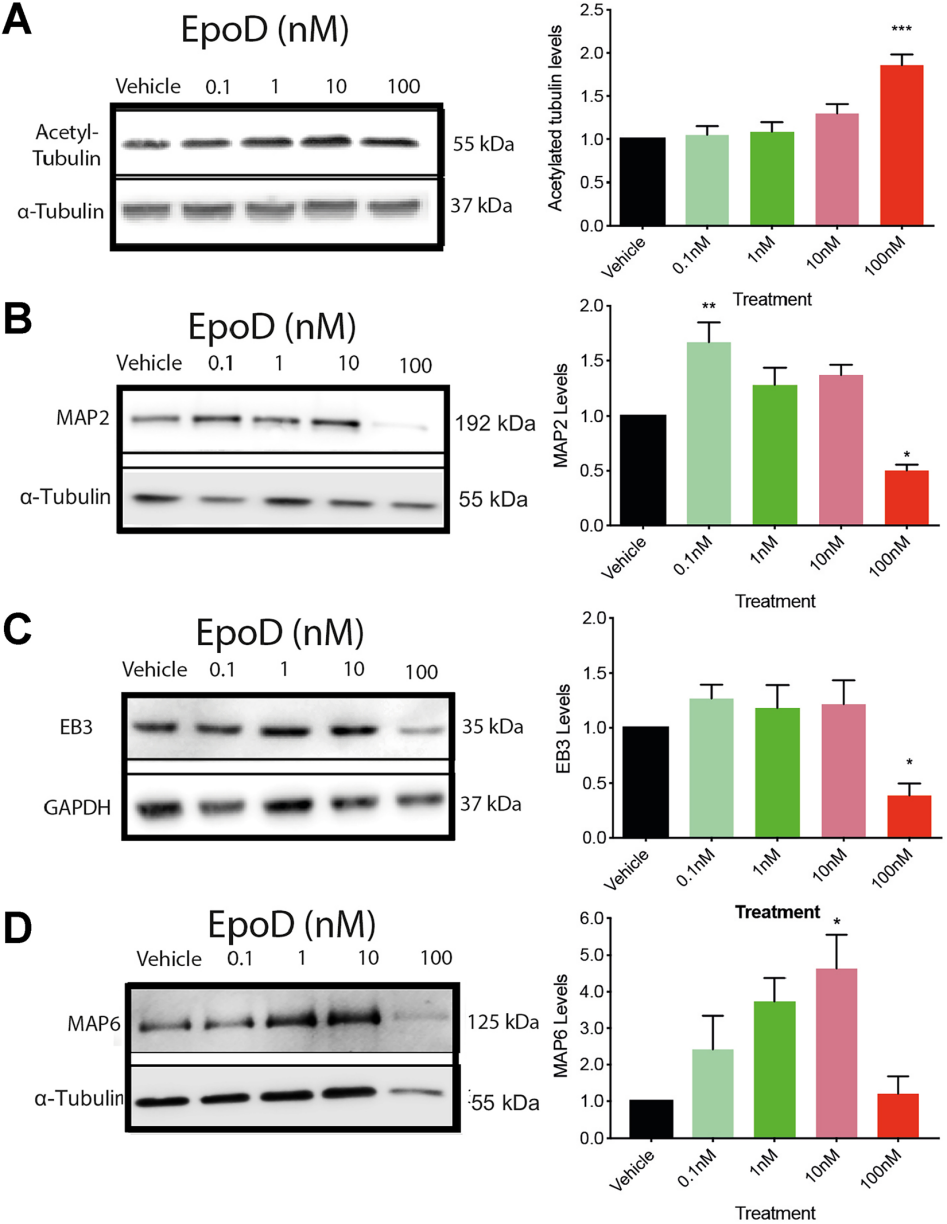
Representative cropped examples of western blot data. (**A**) Tubulin acetylation, a marker of microtubule stability, is increased in 100 nM EpoD treated cortical neuronal cultures. (**B**) Western blots of MAP2 protein levels, (**C**) EB3 protein levels, and (**D**) MAP6 protein levels, compared to GAPDH loading controls. Data reported as mean ± SD, n = 3 cultures, performed in duplicate, ANOVA with Dunnett post hoc test. Abbreviations: DIV, days *in vitro*; EB3, end binding protein; MAP2, microtubule associated protein 2; nM, nanomolar; NFM, neurofilament medium chain; SD, standard deviation; STOP; MAP6, microtubule associated protein 6. Full blot examples are presented in the Supplementary Figure.

Our laboratory^26,43^, and others^44^, have previously used acetylated tubulin levels to measure the stabilising potential of Epothilone D. We have previously reported that cortical neurons treated for two hours with 100 nM of EpoD have markedly increased levels of tubulin acetylation *in vitro*. Indeed, in the current study we treated 10DIV culture cortical neurons with EpoD and evaluated a number of microtubule stability markers and microtubule associated proteins (MAPs). Primary cortical neurons at 10DIV were considered relatively mature, as an increase in synaptic proteins, glutamate receptors, axon/dendrite connections and altered electrophysiological properties occur at this age, and is associated with improved neuronal viability, a necessary phenomenon needed to evaluate any toxicity attributed to by EpoD^45,46^. 100 nM EpoD treated cultures showed significantly (*p* < 0.05) higher levels of acetylation after 24 hours, compared to vehicle treated controls (Fig. 3A). Analysis of specific MAP levels showed that EpoD alters the expression of MAP2, a protein involved in the stabilisation of microtubules, specifically in dendrites, significantly increasing (*p* < 0.01) in 0.1 nM EpoD treated cultures, and a significant decrease in MAP2 protein levels in 100 nM EpoD treated cultures, compared to vehicle treated controls (Fig. 3B). End binding protein 3 (EB3) is a microtubule + TIP protein that is associated with the growing ‘plus’ end of microtubules^47^. Others have reported that microtubule stabilising compounds drastically reduce EB3 expression and localisation^20,21,48^. Evaluation of EB3 protein levels by Western blot reveal 24 hours after treatment EB3 was significantly (*p* < 0.01) decreased in neurons treated with 100 nM of EpoD, with no changes in neurons treated with lower concentrations (Fig. 3C). MAP6, previously known as Stable Tubulin Only Peptide (STOP), is a MAP that is associated with the stable domains of the microtubule network^49^. Treatment of cultures with EpoD revealed that MAP6 levels are significantly (*p* < 0.05) increased in neurons treated with 10 nM of EpoD, compared to vehicle treated control neurons (Fig. 3D). Surprisingly, MAP6 levels in 100 nM EpoD treatments were comparable to vehicle treated controls, most likely due to the loss of neuronal viability (and thus neuronal numbers in the culture). Collectively, MAP analysis suggests that alterations to microtubule protein levels are dose dependent following EpoD treatment.

### Epothilone D alters cellular viability of relatively mature cortical neurons

**Figure 4:**
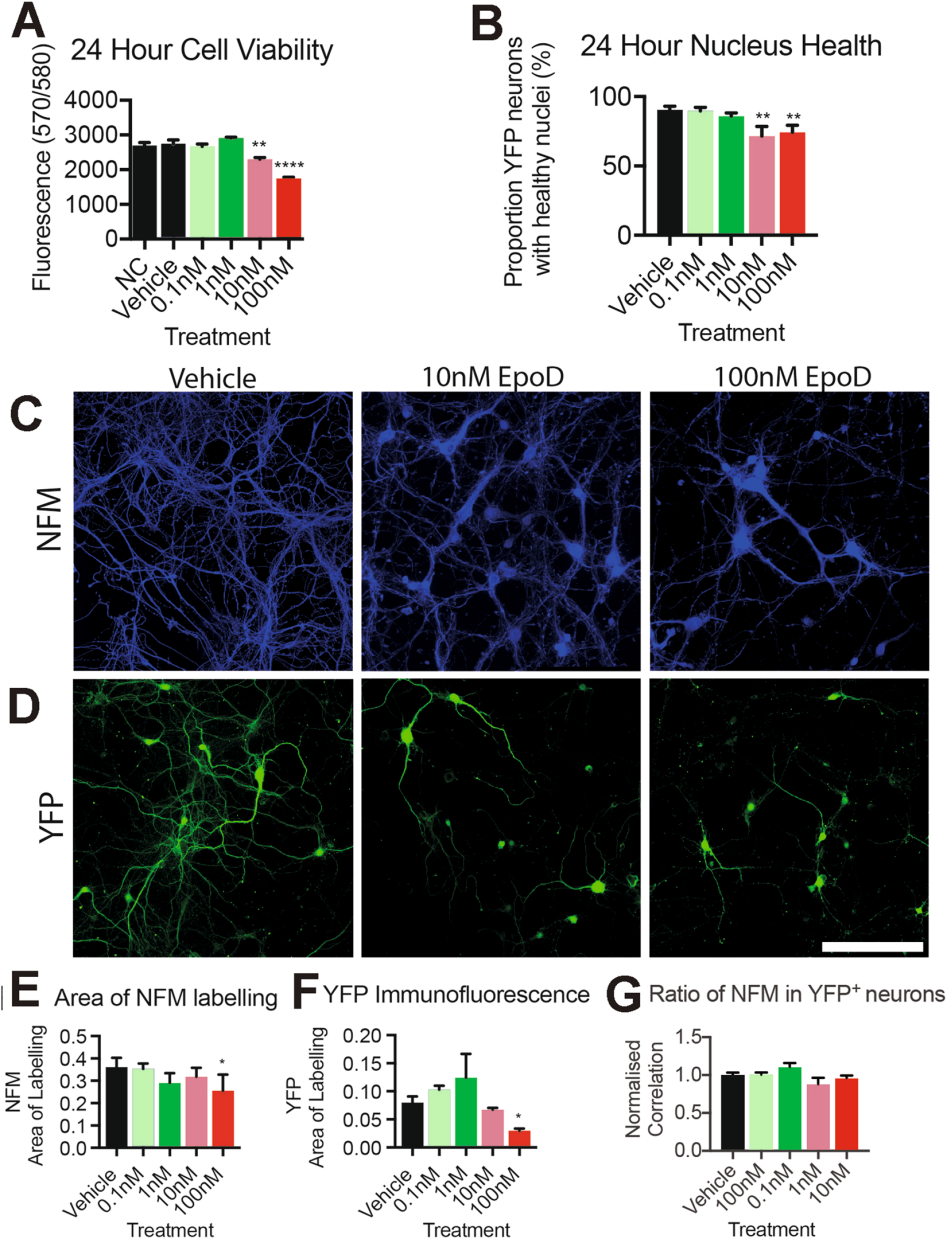
Cellular viability of EpoD treated relatively mature cortical neurons. (**A**) 24 hours post treatment; 10 nM (*p* < 0.05) and 100 nM (*p* < 0.001) concentrations of EpoD are detrimental to cell viability, (**B**) and nuclear morphology (*p* < 0.01), in cortical neurons. (**C**) Neurofilament medium chain (NFM) immunostaining was used as a marker of cytoskeletal and cellular integrity. Treating cortical neurons with 100 nM of EpoD altered the appearance and fluorescence levels of NFM immunostaining, compared to vehicle control treated cultures, and lower EpoD concentrations. (**D**) Endogenously expressed YFP fluorescence appeared reduced in the higher 100 nM EpoD concentration treated cells. Scale = 120 μm. (**E**) The area of NFM immunofluorescence is significantly (*p* < 0.05) reduced in 100 nM EpoD treated cultures. (**F**) The area of YFP immunofluorescence is significantly (*p* < 0.05) decreased in 100 nM EpoD treated cultures. (**G**) The ration of NFM colocalizing with YFP was not significantly changed at any EpoD concentrations. Data reported as mean ± SEM, n = 3 cultures, performed in duplicate, ANOVA with Dunnett post hoc test. Abbreviations: DIV, days *in vitro*; EpoD, epothilone D; nM, nanomolar; NFM, neurofilament medium chain; SEM, standard error of the mean; YFP, yellow fluorescent protein. *p < 0.05, **p < 0.01, ***p < 0.001.

Microtubule targeting compounds, such as EpoD, are generally only used in humans with established cortical structures and circuitry, and typically only in the second and third trimester of pregnant mothers^50^. Therefore, to determine the effect on neurons with established networks, we investigated the impact of EpoD treatment at 10DIV, a stage where cortical neuronal cultures are relatively mature *in vitro*, and neurons begin to develop functional synaptic connections, and have robust viability^26^. Relatively mature primary cortical neurons at 10DIV were treated with varying doses of EpoD and neuronal viability was assessed at 24 hours post-treatment. At 24 hours post-treatment, 10 nM and 100 nM concentrations of EpoD were found to significantly (*p* < 0.05 & 0.001) reduce cell viability by 16% and 36% respectively, compared to vehicle treated controls (Fig. 4A). Similar results were observed upon evaluation of nuclei morphology in YFP expressing neurons at 24 hours post treatment, showing that 10DIV neurons treated with EpoD had a significant (*p* < 0.01) 19% decrease in healthy nuclei in 10 nM treated cultures, and a 16% decrease in 100 nM treated cultures, compared to vehicle treated controls (Fig. 4B). These results suggest that treatment of relatively mature (10DIV) primary cortical neurons with EpoD has dose dependant effects on neuronal viability. Neurofilament medium chain (NFM) immunolabeling was used as a marker of cytoskeletal and cellular integrity. Fluorescence microscopy and subsequent image analysis using thresholding and particle analysis revealed that the area of neurofilament medium chain (NFM) immunolabelling, a neuronal specific cytoskeletal intermediate filament protein, significantly (p < 0.05) decreased in 100 nM EpoD treated cultures (Fig. 4C,E). Similarly, YFP immunofluorescence was significantly decreased (*p* < 0.05) at 100 nM EpoD treatment conditions with no change at lower concentrations (Fig. 4D & F). To determine if the change in NFM reflected a change in neurofilaments or overall cell loss, the amount of NFM in YFP neurons was determined through colocalization analysis. There was no significant differences (*p* > 0.05) in any of the treatments, indicating that NFM expression per se is not effected in the YFP excitatory neurons (Fig. 4G).

### Epothilone D impairs mitochondrial transport in cortical neurons *in vitro*

**Figure 5:**
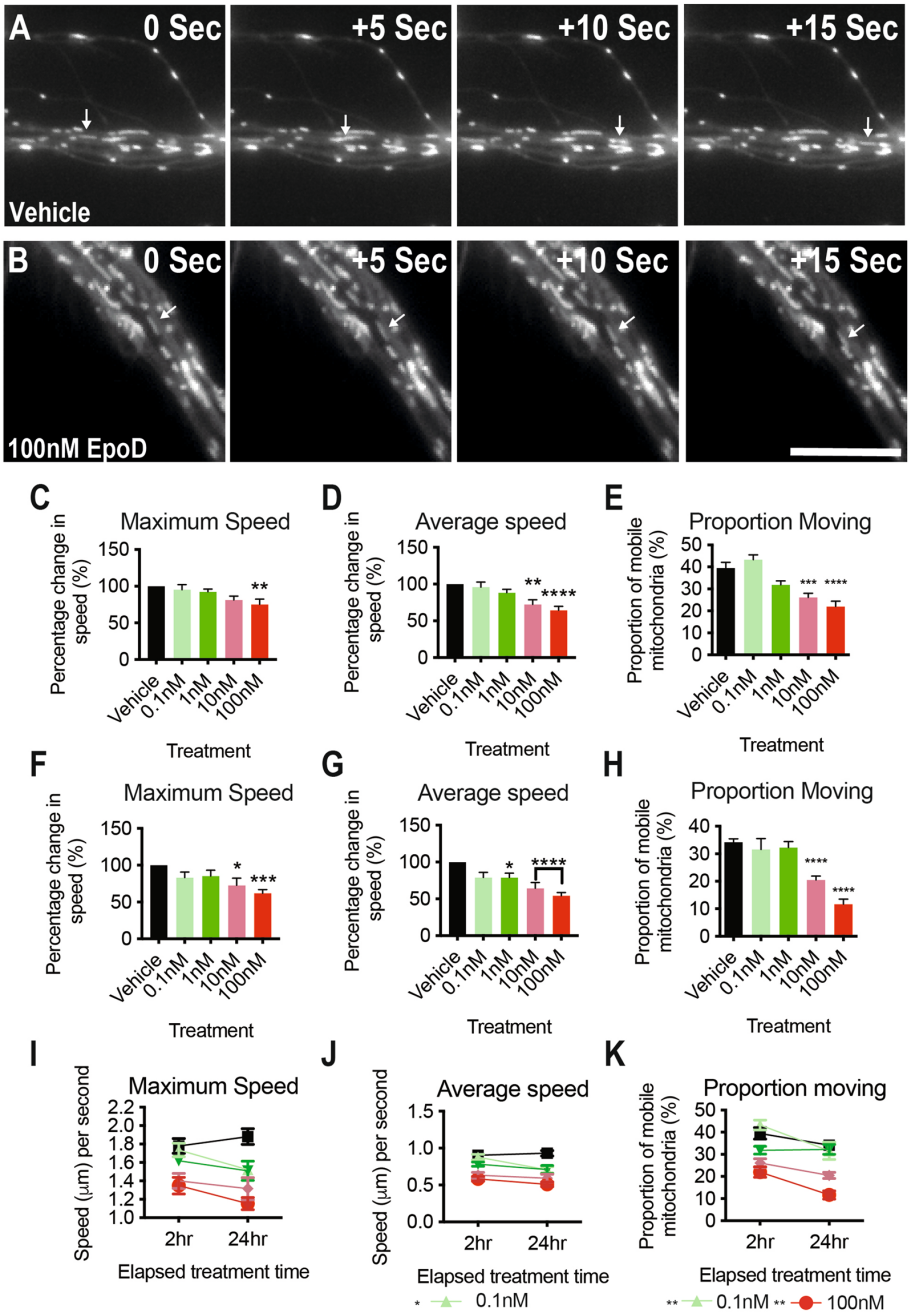
Mitochondrial transport in EpoD treated cortical neurons at 10DIV. (**A**) Mitochondrial transport (arrows) in a vehicle treated axonal bundle, (**B**) and an EpoD treated axonal bundle. (**C**) The maximum speed of mitochondria two hours post EpoD treatment is significantly decreased in 100 nM (*p* < 0.01) treated neuronal cultures. (**D**) Similarly, the average speed of mitochondria two hours post treatment is significantly decreased in 10 nM (*p* < 0.01) and 100 nM (*p* < 0.0001) EpoD treated neuronal cultures. (**E**) The proportion of moving mitochondria is significantly decreased in 10 nM (*p* < 0.001) and 100 nM (p < 0.0001) EpoD treated neuronal cultures. (**F**) The maximum speed of mitochondria 24 hours post EpoD treatment is significantly decreased in 10 nM (*p* < 0.05) and 100 nM (*p* < 0.001) EpoD treated neuronal cultures. (**G**) Similarly, the average speed of mitochondria 24 hours post treatment is significantly decreased in 1 nM (*p* < 0.05), 10 nM (*p* < 0.0001) and 100 nM (*p* < 0.0001) EpoD treated neuronal cultures. (**H**) The proportion of moving mitochondria is significantly (*p* < 0.0001) decreased in 10 nM and 100 nM EpoD treated neuronal cultures 24-hour post treatment. (**I**) The maximum speed of moving mitochondria does not change between two and 24 hour treatments. (**J**) The average speed of mitochondria in 0.1 nM EpoD treated cultures decreased between two hour and 24 hours treated cultures. (**K**) The proportion of moving mitochondria decreased in 0.1 nM and 100 nM EpoD treated cultures between two hours and 24 hours. Data reported as mean ± SEM, n = 10–12 cultures per treatment, performed in triplicate, ANOVA with Dunnett post hoc test. Scale = 50 μm. Abbreviations: DIV, days *in vitro*; nM, nanomolar; SEM, standard error of the mean. *p < 0.05, **p < 0.01, ***p < 0.001, ****p < 0.0001.

Altered microtubule stability has been shown to directly impact microtubule dependant transport in both neurodegenerative diseases, such as Amyotrophic Lateral Sclerosis^51,52^, and during treatment with the microtubule stabilising compound Taxol^21^. Therefore, we hypothesised that treatment of cortical neurons with EpoD would impact mitochondrial transport *in vitro*. Bi-directional microtubule dependant transport was analysed in relatively mature 10DIV cortical neurons by fluorescently labelling mitochondria, followed by live cell imaging. In vehicle treated control cultures, mitochondrial transport is readily observed (Fig. 5A), but is decreased in EpoD treated cultures (Fig. 5B). It was determined that after two hours treatment the maximum transport speed of mitochondria decreases significantly (*p* < 0.01) by 25% in 100 nM EpoD treated cultures, compared to vehicle treated controls (Fig. 5C). Further, the average transport speed of mitochondria two hours after treatment decreased significantly (*p* < 0.001) by 28% in 10 nM EpoD treated neurons and 38% in 100 nM EpoD treated neurons, compared to vehicle treated controls (Fig. 5D). We also evaluated the proportion of moving mitochondria two hours after treatment, with 10 nM EpoD causing a significant (*p* < 0.01) 13% and 100 nM EpoD 18% decrease in the proportion of moving mitochondria in cultured cortical neurons, compared to vehicle treated controls (Fig. 5E).

24 hours post treatment the maximum transport speed of mitochondria was significantly (*p* < 0.05) reduced by 28% in 0.1 nM EpoD treated neurons, and 38% in 100 nM EpoD treated cortical neurons, compared to vehicle treated controls (Fig. 5F). Further, the average transport speed of mitochondria 24 hours after treatment is significantly (*p* < 0.05) decreased by 21% in 1 nM, 36% in 10 nM, and 46% in 100 nM EpoD treated neurons, compared to vehicle treated controls (Fig. 5G). The proportion of moving mitochondria was significantly (*p* < 0.0001) reduced by 14% in 10 nM and 23% in 100 nM EpoD treated neuronal cultures, compared to vehicle treated controls (Fig. 5H). Comparisons of measurement outcomes between the two and 24 hour treatment time points reveal that there was no significant (*p* > 0.05) difference in the maximum speed of mitochondria (Fig. 5I). However, there was a significant (*p* < 0.05) decrease in the average transport speed of mitochondria in 0.1 nM EpoD treated cultures (Fig. 5J). Further, there was a progressive loss (*p* < 0.01) in the proportion of moving mitochondria between the two hour and 24 hour cultures in the 0.1 nM and 100 nM EpoD treated cultures (Fig. 5K). These results suggest that EpoD impairs mitochondrial transport in a dose and time dependent manner, with increasing concentrations having a progressively detrimental effect on transport speed over time. Furthermore, this data indicates that even a sub lethal dose of EpoD, which has no detectable effects on cell health or microtubule dependent neurite outgrowth, can have a significant effect on microtubule dynamics.

## Discussion

Targeting microtubules as a pharmacological intervention is becoming an increasingly attractive and investigated therapeutic strategy for various neurodegenerative diseases and injury^26–29,44^. However, despite the increased use of these CNS penetrant MSAs, the consequence of cortical neuron exposure is still poorly understood^53^. The current study investigated the impact EpoD has on growth, survival and function of cortical neurons *in vitro*, with the aim of evaluating EpoD toxicity in cortical neurons. EpoD was hypothesised to alter microtubule dependant outcomes, such as neurite growth, complexity and length, microtubule stability, microtubule dependant transport and cellular viability in a dose dependant manner. Our study highlights that EpoD impairs various aspects of cortical neuron growth, development and maintenance.

The concentration range of EpoD used in the current study aimed to reflect EpoD concentrations CNS neurons experience as reported in *in vivo* therapeutic trials. It has been suggested that in *in vivo* trials administration of EpoD (1–3 mg/kg) leads to EpoD retainment in the CNS at bioactive levels *in vivo* for days, with cells experiencing low nanomolar concentrations of EpoD^28,38,39,44^. Our results suggest that CNS neurons treated with even relative low doses *in vivo* may be experiencing microtubule dependent dysfunction, particularly when considering organelle transport and general neuronal metabolic activity. Interestingly, treating various types of neurons with EpoB exhibits both beneficial and negative effects depending on neuronal subtype, dosage and the age of the neurons^32^. For example, EpoB was found to decrease cell viability and prevent axonal growth at nanomolar concentrations. However, the authors report that picomolar concentrations of EpoB promoted axonal growth in cortical neurons, a phenomenon not identified in the current study utilising EpoD. Our laboratory has previously shown that 0.1 nM concentrations of EpoD promote axonal regeneration in a scratch assay injury model, supporting the use of sub nanomolar concentrations of EpoD to improve neuronal growth^26^. More recently we have shown a mixture of beneficial and detrimental outcomes follow therapeutic administration of EpoD in an ALS mouse model^43^. Wild type littermates in this study receiving the same dose of EpoD (2 mg/kg) showed no aberrant behavioural, neuronal degeneration or glial activation phenotypes. This suggests that MSAs such as EpoD can have a wide range of effects, which can be dependent upon the disease being targeted, cell types involved and the dosing and timing of such experiments. Indeed, this difficulty in relating dose, treatment timing and innate differences in bioavailability between *in vitro* and *in vivo* models reaffirms that evaluation of differential cellular responses in regard to MSA’s is required to understand the impact of these compounds.

Our study identified that EpoD concentrations similar to those reported in the CNS of EpoD treated mice are neurotoxic to cultured cortical neurons. We investigated cortical neurons at two time points, when they were developing and when then become relative mature *in vitro* at 10 DIV, a time point where the neurons have complex network connections and are synaptically mature. Indeed, EpoD was found to decrease cell viability and growth, and impair microtubule transport, particularly in neuronal cultures treated with high, lethal concentrations of EpoD. Intriguingly, although high concentrations of EpoD are detrimental to neuronal health and the initiation of neurite process extension, our data suggests that large proportions of neurons survive and extend processes, albeit with impacted growth rates and complexity, irrespective of EpoD concentrations. Similar to our study, treatment of cultured sensory neurons with high concentrations of Taxol results in a population of neurons that survive, but with inhibited neurite formation, whereas lower doses of Taxol do not alter survival or neurite outgrowth^54^. Golovyashkina and Colleagues (2015), reported that 0.2 nM of EpoD causes dendritic simplification, whereas higher concentrations cause decreased neuronal survival in cultured CA1 hippocampal neurons^39^. Our data suggests that both sublethal and lethal concentrations of EpoD concentrations cause significant alterations to neurite growth and complexity. Indeed, both axonal and dendritic shortening and simplification occurs to neurons treated with EpoD. Recently we published data showing EpoD administration *in vivo* causes increased dendritic spine density in layer 5 pyramidal projection neurons, without pyramidal cell loss, highlighting the capacity for EpoD to alter different cell types and targets^55^. This difference in EpoD concentration required to cause alterations may suggest a differential vulnerability to EpoD between neuronal populations, similar to that of EpoB, as mentioned previously^32,39^. Further, differences in the vulnerability of neuronal compartments to EpoD were identified in the current study, with only dendrite length being affected at sublethal concentrations, whilst lethal concentrations impacted both axon and dendrite readouts. It would be important in future studies to determine the mechanisms of the differences that may exists between different Epothilones, such and EpoD and EpoB, as our research, in agreement with others is indicating that whilst these two different drugs exhibit similarities, they may have also have very different effects depending upon the cell type and parameter investigated.

We report that EpoD causes dose dependent alterations to the cellular localisation and protein levels of various MAPs. As EpoD concentrations increase, so do markers of stability (acetylation and MAP6), whilst EB3 protein levels decrease only at high, lethal concentrations, suggesting that the microtubule network becomes increasingly stabilised. Both tubulin acetylation and EB3 comets have been previously used as biomarkers for microtubule stability^21,47,48,56^. Pharmacological hyperstabilization of microtubules decreases the density of EB3 + TIPs, thus signifying a reduction in microtubule dynamics and increased microtubule stability^21^. Indeed, a similar phenomenon occurs *in vivo*, where microtubule stabilization in mice suppressed EB3 immunofluorescence in the sciatic nerve^20^ and decreased EB3 comets in the distal axon during phasic pruning^56^. This suggests that microtubule stabilisation with MSAs, such as high, lethal concentrations of EpoD, causes alterations to EB3 protein levels and microtubule + TIP localisation. Interestingly, we identified that MAP6 protein levels are increased at 10 nM concentrations of EpoD, but were reduced back to levels comparable with vehicle controls in 100 nM EpoD treated cultures. The cause of this differential response of MAP6 protein levels is unknown. However, it is quite surprising, as 100 nM EpoD concentrations were shown to increase tubulin acetylation and stability, it would not be unreasonable to assume MAP6 levels would also be increased at this concentration of EpoD. MAP6, a protein that binds to microtubules in response to cold induced depolymerisation^57^, can be regulated by calmodulin^58,59^, and is susceptible to increasing intracellular calcium levels^60^. The decrease in MAP6 levels in 100 nM EpoD treated cultures in the current study may be explained by an increase in intracellular calcium due to decreased cellular viability^60^. Interestingly, MAP6-null mice are used as a model of schizophrenia, as microtubules have been implicated in the disorder^30,31,61^. EpoD treatment of MAP6-null mice improves this disorder by stabilising microtubules^31^, suggesting the stability properties of both MAP6 and EpoD affect stable microtubule domains. Investigations into whether EpoD administration causes aberrant MAP phenotypes, such as Benbow and Colleagues (2016) described when treating mice with taxol and describing changes to EB3 expression *in vivo*, is required to compare our current study with EpoD concentrations used for cancer therapy^62^. Whilst we identified the majority of changes to MAP levels occur only at lethal concentrations of EpoD, and in keeping with this MAP2 levels were significantly reduced at the lethal 100 nM concentration, we also identified that MAP2 levels were in fact increased at the sublethal concentration with 0.1 nM. This suggests that MAP2, a dendrite specific MAP, may have an differential response to a range of EpoD concentrations, supported by the identified changes to dendrite length at sublethal concentrations of EpoD, that may be titrated.

Altered microtubule stability and dynamics can also impact microtubule dependant intracellular transport^21,48^, a phenomenon associated with pathophysiological mechanism in neurodegenerative disorders, such as Motor Neuron Disease^51,52^. Our results suggest that whilst low, sublethal concentrations and short treatment durations with EpoD may not impact neuronal survival and viability, subtle changes to bidirectional mitochondrial transport occur, with increasing concentrations having a progressively detrimental effect on transport speed over time. Indeed, whilst sublethal concentrations of EpoD only impact the average transport speed of mitochondria after 24 hours of treatment, lethal concentrations of EpoD impact all mitochondrial readouts, regardless of treatment duration. Correct mitochondrial transport is imperative for neuronal survival^63^ and mitochondrial transport is important for axonal and dendrite growth^64,65^, which may explain the alterations in both growth and morphology identified in our study. Indeed, we focused on pooling analysis of axons and dendrites in our mitochondrial transport analysis, therefore we cannot comment directly whether anterograde transport, retrograde transport, or both are affected by EpoD treatment. Furthermore, it was not determined if this altered transport was also associated with altered function. Our viability assays, which employed the Alamar Blue assay, would be affected by mitochondrial dysfunction but this cannot be interoperated as a change in mitochondrial function alone. Taken together with aberrant neuronal development during exposure to low EpoD concentrations, we conclude that EpoD has wide and far-reaching effects in regard to dosage and duration of treatment.

## Conclusion

Microtubule targeting compounds are increasingly being considered for their therapeutic potential in various neurodegenerative diseases. However, how these compounds influence normal neuronal functioning is still poorly understood. Our findings have important implications for both the proposed therapeutic use of low dose MSAs in the treatment of neurodegeneration and disease, and informing the mechanisms by which higher doses may provoke peripheral neuropathies. It was found that EpoD impaired normal neuronal growth and viability and caused dysfunction to microtubule dependant transport, at concentrations comparable to those used in previous *in vivo* preclinical neurodegenerative trials. It was found that the majority of effects on mitochondrial movement are caused by high, lethal concentrations of EpoD. However, our data also suggested that low sublethal concentrations of EpoD may affect average transport speed of mitochondria over time. These findings suggest that protracted periods of seemingly mild EpoD exposure may have consequences on mitochondrial function and that consideration of dose of EpoD, as well as the intended target or outcome are essential when utilising EpoD as a pharmacological modifier of neurodegenerative diseases and trauma. Comparisons between chronic and acute exposure, the bioavailability of MSAs to distinct anatomical and cellular compartments, the differential vulnerability of a range of cell populations to MSAs, and systemic versus localised exposure during treatment are required. This is of particular importance where the microtubule network may be differentially altered in injury and disease states, thus making both timing and dose highly relevant. It is essential to understand the underlying impact of MSA action on cellular function, particularly when MSAs continue to be a possible avenue of therapeutic intervention for multiple neurological disorders. Future studies utilising pharmacological manipulation of microtubule stability would benefit from a greater understanding of the microtubule environment, and how individual MSAs affect the cytoskeleton.

## Supplementary information

Dataset 1.

## References

[1] Cameron HA, Woolley CS, McEwen BS, Gould E (1993). Differentiation of newly born neurons and glia in the dentate gyrus of the adult rat. Neuroscience.

[2] Gonzalez-Perez O (2012). Neural stem cells in the adult human brain. Biol. Biomed. Rep..

[3] Kole K, Scheenen W, Tiesinga P, Celikel T (2018). Cellular diversity of the somatosensory cortical map plasticity. Neurosci. Biobehav. Rev..

[4] Dent EW (2017). Of microtubules and memory: implications for microtubule dynamics in dendrites and spines. Mol. Biol. Cell.

[5] Baas PW, Black MM (1990). Individual microtubules in the axon consist of domains that differ in both composition and stability. J. Cell Biol..

[6] Baas PW, Rao AN, Matamoros AJ, Leo L (2016). Stability properties of neuronal microtubules. Cytoskeleton.

[7] Mitchison T, Kirschner M (1984). Dynamic instability of microtubule growth. Nature.

[8] van Beuningen SF (2015). TRIM46 controls neuronal polarity and axon specification by driving the formation of parallel microtubule arrays. Neuron.

[9] Allen C, Borisy GG (1974). Structural polarity and directional growth of microtubules of Chlamydomonas flagella. J. Mol. Biol..

[10] Chen BL, Hall DH, Chklovskii DB (2006). Wiring optimization can relate neuronal structure and function. Proc. Natl Acad. Sci. USA.

[11] Baas PW, Lin S (2011). Hooks and comets: the story of microtubule polarity orientation in the neuron. Dev. Neurobiol..

[12] Sakakibara A, Ando R, Sapir T, Tanaka T (2013). Microtubule dynamics in neuronal morphogenesis. Open. Biol..

[13] Nakata T, Niwa S, Okada Y, Perez F, Hirokawa N (2011). Preferential binding of a kinesin-1 motor to GTP-tubulin-rich microtubules underlies polarized vesicle transport. J. Cell Biol..

[14] Pacheco A, Gallo G (2016). Actin filament-microtubule interactions in axon initiation and branching. Brain Res. Bull..

[15] Dent EW, Kalil K (2001). Axon branching requires interactions between dynamic microtubules and actin filaments. J. Neurosci..

[16] Dumontet C, Jordan MA (2010). Microtubule-binding agents: a dynamic field of cancer therapeutics. Nat. Rev. Drug. Discov..

[17] Zhou J, Giannakakou P (2005). Targeting microtubules for cancer chemotherapy. Curr. Med. Chem. Anticancer. Agents.

[18] Lee JJ, Swain SM (2006). Peripheral neuropathy induced by microtubule-stabilizing agents. J. Clin. Oncol..

[19] Carlson K, Ocean AJ (2011). Peripheral neuropathy with microtubule-targeting agents: occurrence and management approach. Clin. Breast Cancer.

[20] Benbow SJ (2016). Effects of paclitaxel and eribulin in mouse sciatic nerve: a microtubule-based rationale for the differential induction of chemotherapy-induced peripheral neuropathy. Neurotox. Res..

[21] Shemesh OA, Spira ME (2010). Paclitaxel induces axonal microtubules polar reconfiguration and impaired organelle transport: implications for the pathogenesis of paclitaxel-induced polyneuropathy. Acta Neuropathol..

[22] Gu J, Zheng JQ (2009). Microtubules in dendritic spine development and plasticity. Open. Neurosci. J..

[23] Fanara P (2010). Changes in microtubule turnover accompany synaptic plasticity and memory formation in response to contextual fear conditioning in mice. Neuroscience.

[24] Eira J, Silva CS, Sousa MM, Liz MA (2016). The cytoskeleton as a novel therapeutic target for old neurodegenerative disorders. Prog. Neurobiol..

[25] Bollag DM (1995). Epothilones, a new class of microtubule-stabilizing agents with a taxol-like mechanism of action. Cancer Res..

[26] Brizuela M (2015). The microtubule-stabilizing drug Epothilone D increases axonal sprouting following transection injury *in vitro*. Mol. Cell Neurosci..

[27] Cartelli D (2013). Microtubule alterations occur early in experimental parkinsonism and the microtubule stabilizer epothilone D is neuroprotective. Sci. Rep..

[28] Brunden KR (2010). Epothilone D improves microtubule density, axonal integrity, and cognition in a transgenic mouse model of tauopathy. J. Neurosci..

[29] Zhang B (2012). The microtubule-stabilizing agent, epothilone D, reduces axonal dysfunction, neurotoxicity, cognitive deficits, and Alzheimer-like pathology in an interventional study with aged tau transgenic mice. J. Neurosci..

[30] Andrieux A (2006). Microtubule stabilizer ameliorates synaptic function and behavior in a mouse model for schizophrenia. Biol. Psychiatry.

[31] Fournet V (2012). Both chronic treatments by epothilone D and fluoxetine increase the short-term memory and differentially alter the mood status of STOP/MAP6 KO mice. J. Neurochem..

[32] Jang EH, Sim A, Im SK, Hur EM (2016). Effects of Microtubule Stabilization by Epothilone B Depend on the Type and Age of Neurons. Neural Plast..

[33] Konner J (2012). Phase I clinical, pharmacokinetic, and pharmacodynamic study of KOS-862 (Epothilone D) in patients with advanced solid tumors and lymphoma. Invest. N. Drugs.

[34] Feng G (2000). Imaging neuronal subsets in transgenic mice expressing multiple spectral variants of GFP. Neuron.

[35] Kihlmark M, Imreh G, Hallberg E (2001). Sequential degradation of proteins from the nuclear envelope during apoptosis. J. Cell Sci..

[36] Schneider CA, Rasband WS, Eliceiri KW (2012). NIH Image to ImageJ: 25 years of image analysis. Nat. Methods.

[37] Meijering E, Dzyubachyk O, Smal I (2012). Methods for cell and particle tracking. Methods Enzymology.

[38] Albright, C. F., Barten, D. M. & Lee, F. Y. (Google Patents, 2011).

[39] Golovyashkina N (2015). Region-specific dendritic simplification induced by Abeta, mediated by tau via dysregulation of microtubule dynamics: a mechanistic distinct event from other neurodegenerative processes. Mol. Neurodegeneration.

[40] Witte H, Neukirchen D, Bradke F (2008). Microtubule stabilization specifies initial neuronal polarization. J. Cell Biol..

[41] Kapitein LC, Hoogenraad CC (2015). Building the Neuronal Microtubule Cytoskeleton. Neuron.

[42] Lewis TL, Courchet J, Polleux F (2013). Cell biology in neuroscience: cellular and molecular mechanisms underlying axon formation, growth, and branching. J. Cell Biol..

[43] Clark J. A., Blizzard C. A., Breslin M. C., Yeaman E. J., Lee K. M., Chuckowree J. A., Dickson T. C. (2018). Epothilone D accelerates disease progression in the SOD1G93Amouse model of amyotrophic lateral sclerosis. Neuropathology and Applied Neurobiology.

[44] Brunden KR (2011). The characterization of microtubule-stabilizing drugs as possible therapeutic agents for Alzheimer’s disease and related tauopathies. Pharmacol. Res..

[45] Wang W (2008). Electrophysiological properties of mouse cortical neuron progenitors differentiated *in vitro* and *in vivo*. Int. J. Clin. Exp. Med..

[46] Lesuisse C, Martin LJ (2002). Long-term culture of mouse cortical neurons as a model for neuronal development, aging, and death. J. Neurobiol..

[47] Dent EW, Baas PW (2014). Microtubules in neurons as information carriers. J. Neurochem..

[48] Kleele T (2014). An assay to image neuronal microtubule dynamics in mice. Nat. Commun..

[49] Bosc C (1996). Cloning, expression, and properties of the microtubule-stabilizing protein STOP. Proc. Natl Acad. Sci. USA.

[50] Zheng X, Zhu Y, Zhao Y, Feng S, Zheng C (2017). Taxanes in combination with platinum derivatives for the treatment of ovarian cancer during pregnancy: a literature review. Int. J. Clin. Pharmacol. Ther..

[51] Bilsland LG (2010). Deficits in axonal transport precede ALS symptoms *in vivo*. Proc. Natl Acad. Sci. USA.

[52] Fanara P (2007). Stabilization of hyperdynamic microtubules is neuroprotective in amyotrophic lateral sclerosis. J. Biol. Chem..

[53] Baas PW, Ahmad FJ (2013). Beyond taxol: microtubule-based treatment of disease and injury of the nervous system. Brain.

[54] Letourneau PC, Ressler AH (1984). Inhibition of neurite initiation and growth by taxol. J. Cell Biol..

[55] Chuckowree JA (2018). The microtubule-modulating drug epothilone D alters dendritic spine morphology in a mouse model of mild traumatic brain injury. Front. Cell. Neurosci..

[56] Brill Monika S., Kleele Tatjana, Ruschkies Laura, Wang Mengzhe, Marahori Natalia A., Reuter Miriam S., Hausrat Torben J., Weigand Emily, Fisher Matthew, Ahles Andrea, Engelhardt Stefan, Bishop Derron L., Kneussel Matthias, Misgeld Thomas (2016). Branch-Specific Microtubule Destabilization Mediates Axon Branch Loss during Neuromuscular Synapse Elimination. Neuron.

[57] Slaughter T, Black MM (2003). STOP (stable-tubule-only-polypeptide) is preferentially associated with the stable domain of axonal microtubules. J. Neurocytol..

[58] Bosc C (2001). Identification of novel bifunctional calmodulin-binding and microtubule-stabilizing motifs in STOP proteins. J. Biol. Chem..

[59] Lefevre J (2013). Structural basis for the association of MAP6 protein with microtubules and its regulation by calmodulin. J. Biol. Chem..

[60] Job D, Fischer EH, Margolis RL (1981). Rapid disassembly of cold-stable microtubules by calmodulin. Proc. Natl Acad. Sci. USA.

[61] Volle J (2013). Reduced expression of STOP/MAP6 in mice leads to cognitive deficits. Schizophr. Bull..

[62] Cheng KL, Bradley T, Budman DR (2008). Novel microtubule-targeting agents - the epothilones. Biologics.

[63] Sheng ZH, Cai Q (2012). Mitochondrial transport in neurons: impact on synaptic homeostasis and neurodegeneration. Nat. Rev. Neurosci..

[64] Athamneh AIM (2017). Neurite elongation is highly correlated with bulk forward translocation of microtubules. Sci. Rep..

[65] Nguyen HTN (2018). Impaired neurite development associated with mitochondrial dysfunction in dopaminergic neurons differentiated from exfoliated deciduous tooth-derived pulp stem cells of children with autism spectrum disorder. Biochem. Biophys. Rep..

