# Supplementary material for: Epothilone D alters normal growth, viability and microtubule dependent intracellular functions of cortical neurons in vitro
Source: Sci Rep. 2020 Jan 22;10:918. doi: 10.1038/s41598-020-57718-z (PMC6976590; doi:10.1038/s41598-020-57718-z)
Supplement: Supplementary file 1 — Dataset 1. [file 41598_2020_57718_MOESM1_ESM.pdf]

Epothilone D alters normal growth, viability and microtubule dependent intracellular functions of cortical neurons *in vitro*.

J. A. Clark, J. A. Chuckowree, M. S. Dyer, T. C. Dickson, and C. A. Blizzard.

Supplementary figure and methods for western blots used for quantification.

Complete western blots of data presented in Figure 3. (A) Acetylated tubulin blot, (B) MAP2 blot, arrow denotes line quantified, (C) EB3 blot and (D) MAP6 blot.

Methods: Neuronal cultures were rinsed in ice-cold PBS, followed by lysis in RIPA-Inhibition buffer (RIPA buffer + 1µM trichostatin-a (Sigma Aldrich) + protease inhibitor cocktail (Sigma Aldrich)), and centrifugation at 15,000g for 10 minutes at 4°C to remove cell debris, as described previously [28]. Proteins were separated by SDS-PAGE using a Novex NuPAGE gel system (Thermo Fisher Scientific), followed by probing by Western blot. Protein levels were then evaluated using antibodies to acetylated tubulin (mouse-monoclonal, 1:5000, Sigma Aldrich), alpha-tubulin (mouse-polyclonal, 1:5000, Abcam), MAP2 (mouse-monoclonal, 1:1000, Millipore, MAB3418), MAP6/STOP (mouse-monoclonal, 1:1000, Millipore, MAB5524) and EB3 (rat-monoclonal, 1:200, Abcam, AB53360), with GAPDH (rabbit polyclonal, 1:5000, Millipore) and alpha-tubulin (mouse-monoclonal, 1:5000, Abcam) used as loading controls. Secondary HRP conjugated antibodies (DAKO anti-mouse and rabbit, 1:5000; Invitrogen anti-rat, 1:2000) were incubated for 1.5 hours, followed by rinsing with TBS-T. Membranes were then probed by incubating with Immobilon chemiluminescence HRP substrate (Millipore) for 5 mins, followed by detection with Chemi-Smart 5000 image station (Vilber Lourmat). Band quantitation was completed using densitometry analysis in ImageJ. Blot stripping was completed using Restore Western Blot Stripping Buffer (Thermo Fisher Scientific, 21059).

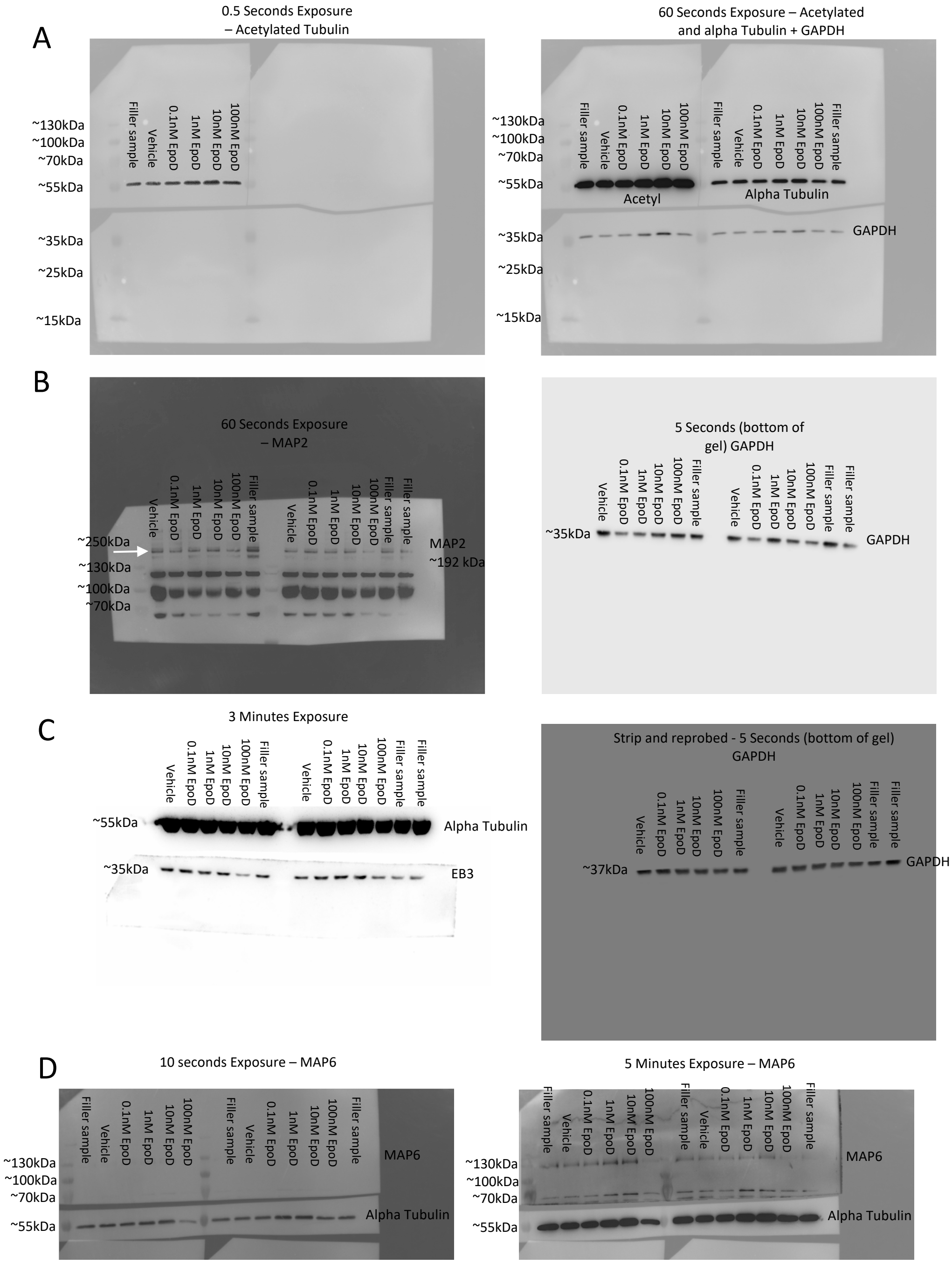
A
0.5 Seconds Exposure – Acetylated Tubulin
Filler sample
Vehicle
0.1nM EpoD
1nM EpoD
10nM EpoD
100nM EpoD
~130kDa
~100kDa
~70kDa
~55kDa
~35kDa
~25kDa
~15kDa
60 Seconds Exposure – Acetylated and alpha Tubulin + GAPDH
Acetyl
Alpha Tubulin
GAPDH
B
60 Seconds Exposure – MAP2
~250kDa
MAP2 ~192 kDa
5 Seconds (bottom of gel) GAPDH
Filler sample
C
3 Minutes Exposure
Alpha Tubulin
EB3
Strip and reprobed - 5 Seconds (bottom of gel) GAPDH
~37kDa
D
10 seconds Exposure – MAP6
MAP6
5 Minutes Exposure – MAP6
